# The Need of Idiot Asylums

**Published:** 1899-01-07

**Authors:** 


### THE NEED OF IDIOT ASYLUMS.

In our story of the insane we have more than once referred to the advantages which would accrue if all our county asylums were to provide annexes for the care and treatment of idiot children; and our attention has been recently drawn to the subject by an interested correspondent.

The idiot asylums at Earlswood, Colchester, and Lancaster, as a rule, receive children for a limited number of years only. It consequently sometimes happens that the idiot is returned to the parents little, if at all, improved; and then a fresh residence must be found elsewhere, or a very undesirable member of the family retained.

So far only four county asylums are provided with these useful annexes, namely, Hampshire, Northamptonshire, Warwickshire, and Surrey, at Wandsworth; and there is the Metropolitan Asylum for Idiots at Darenth. In these places, when the idiot becomes too old for retention in the schools and not well enough for discharge to his home, he is merely transferred to the adult part of the house, and the age difficulty is got rid of without difficulty.

That such adjuncts should be part of the equipment of every county asylum we think can hardly admit of a second opinion, inasmuch as the wants of the county can be provided for at the same time that profits are made from the private patients from their own or other districts. County Councils should have the urgency of this question pointed out to them, so that they may instruct their Asylum Committees to set about the work of building annexes ranging from 40 to 100 beds.

In the meantime we should advise our correspondent to apply at the asylums we have named, and he will most likely find accommodation in one of them at 20s. a week or less. If the sum asked be above his means, we would further point out to him that the Lunacy Act specially provides for such cases as his, for it is enacted that any lunatic, idiot, or person of unsound mind, if in receipt of relief, or who may need relief for his proper treatment, may be sent to the asylum of his own county.

All that is necessary, then, is to call in the relieving officer, and he is bound to take immediate steps for the removal of the patient. The only possible obstacle to this removal would be want of room in the county asylum, and this want, if it exists, might necessitate the authorities to send the idiot to the asylum of some other county; and if one of the above-named counties be selected our correspondent will obtain what he wants.

